# Barriers to and Facilitators of Phosphate Control in Children With CKD

**DOI:** 10.1016/j.ekir.2025.09.045

**Published:** 2025-10-10

**Authors:** Louise McAlister, Vanessa Shaw, Pearl Pugh, Triona Joyce, Evelien Snauwaert, Fionna Bathgate, Charlotte Holt, Caroline Anderson, An Desloovere, José Renken-Terhaerdt, Maria Rosa Grassi, Sevcan Bakkaloğlu, Gulsah Sahin, Rukshana Shroff, Kelly Lambert

**Affiliations:** 1 Great Ormond Street Hospital for Children NHS Foundation Trust, London, UK; 2 UCL Great Ormond Street Institute of Child Health, University College London, UK; 3 Nottingham Children’s Hospital, Nottingham University Hospitals NHS Trust, Nottingham, UK; 4 Department of Nutrition and Dietetics, Guy’s & St Thomas’ NHS Foundation Trust, Westminster Bridge Road, London, UK; 5 Department of Pediatric Nephrology, Ghent University Hospital, Ghent, Belgium; 6 University Hospital Southampton NHS Foundation Trust, Southampton, UK; 7 University Hospital Ghent, Ghent, Belgium; 8 Wilhelmina Children’s Hospital, University Medical Center Utrecht, Utrecht, The Netherlands; 9 University of Milan, Milan, Italy; 10 Gazi University, Ankara, Turkey; 11 Atılım University, Ankara, Turkey; 12 University of Wollongong, Wollongong, New South Wales, Australia

**Keywords:** adherence, children, chronic kidney disease, diet, phosphate-binders, focus group

## Abstract

**Introduction:**

Managing mineral and bone disorder in children with chronic kidney disease (CKD) requires control of serum phosphate levels. However, hyperphosphatemia is common, particularly in adolescents, reflecting suboptimal adherence to phosphate-binder medications and a reduced phosphate diet. We explored phosphate-related knowledge and adherence barriers in children, and their caregivers, using a sequential explanatory mixed-methods study design.

**Methods:**

Children aged 8 to 18 years with CKD stages 4 and 5, on dialysis or post-transplantation, and caregivers, were recruited from 3 UK pediatric kidney centers. The Phosphate Understanding and Knowledge Assessment questionnaire was used to assess knowledge. Online focus groups explored real-world challenges to phosphate control.

**Results:**

Forty-eight children and 43 caregivers were recruited; 44 (92%) children and 33 (75%) caregivers completed the questionnaire. Median knowledge scores were 64.3% (interquartile range, 55.3–78.6) for children and 72.7% (interquartile range, 64.3–85.7) for caregivers (*P* = 0.04). Older children scored higher (*P* = 0.01, *R*^2^ = 0.13), but knowledge did not correlate with serum phosphate. Dietary restriction was perceived as more challenging than using phosphate-binders (59% children; 71% caregivers). Forty-six participants, including 30 child-caregiver dyads, joined focus groups. The following 5 themes were identified encapsulating the experiences of families: practical advice and support are valued; personalized strategies are preferred to facilitate sense-making; the social environment of the child and family is disrupted; education and self-management skills can influence success; and the journey requires acceptance, adaptation, and perseverance.

**Conclusions:**

In pediatric CKD, poor adherence to phosphate advice originates more from social and practical barriers than knowledge deficits. Our findings can inform personalized strategies to improve adherence in real-world settings.

## Graphical abstract

**Figure:**
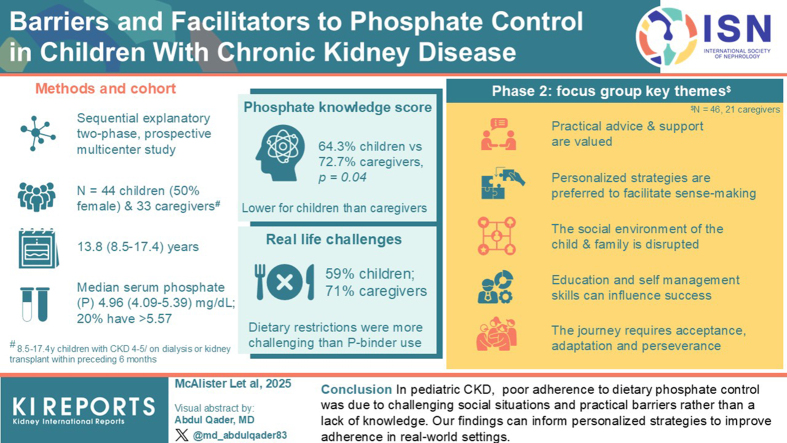

Prevention and control of mineral and bone disorder is a key concern in children with CKD. Reducing dietary phosphate intake^1^ and using phosphate binder (P-binder) medications are critical interventions. However, adherence to diet and P-binders is reported to be < 50% in adults^2^^,^^3^ and similarly low in children.^4^ Hyperphosphatemia has been reported in nearly half of children on dialysis, especially adolescents,^5^ putting them at risk of adverse bone and cardiovascular health outcomes.

Contemporary education for hyperphosphatemia management focuses on increasing practical knowledge and skills regarding dietary-phosphate reduction (from both phosphate-based additives and naturally occurring phosphate)^6^ and appropriate distribution of P-binders. However, existing educational materials are often confusing, may present conflicting messages, and are primarily targeted at caregivers.^7^^,^^8^ It has been suggested that children may require innovative and age-appropriate educational approaches to support self-management.^9^ To our knowledge, no previous research has specifically examined how children and their caregivers engage with dietary and medical phosphate advice, or the challenges they face adhering to the prescribed treatments.

To address this gap in knowledge, the Improving Phosphate Control in Children With CKD (IMPACT) study explored phosphate-related knowledge, educational preferences, barriers to or facilitators of adherence to dietary-phosphate control, and the use of P-binders in children with CKD and their caregivers.

## Methods

A sequential explanatory 2-phase, prospective multicenter study was designed. Phase 1 included questionnaire completion and phase 2 comprised qualitative feedback via online focus groups (Supplementary Figure S1). Ethical approval was granted by the Health Research Authority and Care Research Wales on November 1, 2022 - reference: 22/SW/0150. This study is registered on clinicaltrials.gov (NCT05439980).

### Participant Selection

Children aged between 8 and 18 years with CKD stages 4 and 5, on dialysis or those with kidney transplantation within the preceding 6 months, and their caregivers, were recruited from 3 kidney units in the UK. Children with neurodevelopmental or cognitive disorders, underlying phosphate-losing tubular disorders, exclusive enteral tube-feeding or limited spoken or written language skills were excluded. Recruitment to focus groups ceased when data saturation was reached and no new concepts or experiences were described.^10^

Potential participants were identified from hospital records. Informed consent and assent from children and caregivers was obtained for both phases. Demographics and routine biochemistry, from the most recent clinic review (or from the midweek blood sampling for children on hemodialysis), were recorded.

### Questionnaire Design

Two questionnaires were designed specifically for this study: the Phosphate Understanding and Knowledge Assessment questionnaires for children or adults (PUKA-c and PUKA-a) with 36 and 37 questions respectively. The development of these questionnaires has been documented in an earlier publication.^11^ Thirteen questions were knowledge-based, with a potential score of 14 (or 11 if not taking P-binders), with scoring expressed as percentage correct.

### Focus Groups

Online focus groups explored experiences of phosphate management and facilitated the sharing of personal perspectives.^12^^,^^13^ Separate 1-hour focus groups of 2 to 6 children or caregivers were moderated by the principal investigator (LM), with field notes taken by a member of the research team (PP, CH, or FB). The principal investigator was known by some, but not all, participants. A topic guide was created to aid discussions (Supplementary Figure S2) and adapted iteratively as the focus groups progressed. Participants were shown resources currently used in phosphate education to stimulate discussion about their education experiences. All participants were invited to articulate advice they would give to other children (or caregivers) on phosphate management. The focus groups were audio-recorded with permission, and transcribed verbatim. Results were reported in accordance with the Consolidated Criteria for Reporting Qualitative Studies.^14^

Data were analyzed using inductive thematic analysis,^15^^,^^16^ involving familiarization, coding, generating; and subsequently reviewing, refining, and defining themes. Transcripts were inductively coded line by line by LM, KL, VS, PP, FB, and CH. Codes were then grouped into categories and aggregated into themes and subthemes. Exemplar quotes were extracted for each theme and tabulated for examination. Investigator triangulation of preliminary themes was undertaken to reduce bias and strengthen credibility. The final themes were agreed by key members of the research team (LM, KL, RS, VS, PP, FB, and CH) and shared with the participants who confirmed they were representative of their experiences.

The results from phases 1 and 2 were used to derive actionable strategies for clinicians to facilitate dietary adherence.

### Statistical Analysis

Data were analyzed using RStudio (R version 4.3.2). Continuous data are reported using mean and SD or median and interquartile range. Cronbach’s alpha analysis was used to assess the reliability and internal consistency of the knowledge items in the PUKA questionnaire.^17^ Pearson’s correlation measured the relationship between knowledge scores with serum phosphate levels, CKD stage, age, sex and P-binder dose. Sex was defined by biological traits such as chromosomes, anatomy and hormone levels. Statistical significance was set at an an alpha level of 0.05.

## Results

A total of 91 participants (48 children and 43 caregivers) were recruited. Seventy-seven participants completed the questionnaires (44 children and 33 caregivers, 85% response rate), including 30 child-caregiver dyads. Three children and 14 caregivers declined to complete the questionnaire, citing lack of time or, for caregivers, no longer being actively involved in their child’s care. The median age of pediatric participants was 13.8 (range: 8.5–17.4) years, with 22 (50%) female. English was the dominant language in ≥ 80% with 22 (50%) identifying as being from an ethnic minority background. P-binders were taken by > 75% of the children (Table 1). The median serum-phosphate was 4.96 mg/dl (interquartile range: 4.09–5.39, Table 2) with 20% of participants having a serum phosphate > 5.57 mg/dl. Older children (aged ≥ 13 years) had a significantly higher serum- phosphate (*P* = 0.01) compared with those aged < 13 years. These age group categories were chosen by the study team to reflect potential differences between children and adolescents in terms of development and adherence.

**Table 1:** Demographics of study population

Variable, *n*		Questionnaire (PUKA)		Focus groups	
Respondents	Child	44		25	
	Caregivers	33		21 (4 M, 17 F)	
	Child-caregiver dyads	30			
Variable, *n* (%)		Child	Caregiver	Child	Caregiver
Centre	GOSH	30 (68)	20 (61)	18 (72)	13 (62)
	ECH	10 (23)	11 (33)	4 (16)	6 (29)
	NCH	4 (9)	2 (6)	3 (12)	2 (9)
Age (Child)	8 to < 13 yrs	15 (34)	13 (39)	8 (32)	9 (43)
	13–18 yrs	29 (66)	20 (61)	17 (68)	12 (57)
Sex (Child)	Girl	22 (50)	18 (55)	12 (49)	11 (52)
Ethnicity	African	5 (11)	3 (9)	2 (8)	-
	Asian	8 (18)	5 (15)	4 (16)	3 (14)
	White	22 (50)	19 (58)	15 (60)	14 (67)
	Mixed	8 (18)	6 (18)	3 (12)	4 (19)
	Other	1 (2)	0	1 (4)	-
Main language	English	42 (96)	32 (97)	25 (100)	21 (100)
(Child)	Urdu	1 (2)	1 (3)	-	-
	Polish	1 (2)	-	-	-
Main language	English	35 (80)	28 (85)	21 (84)	20 (95)
Spoken at home	Other^a^	9 (20)	5 (15)	4 (16)	1 (5)
Kidney disease	CAKUT	24 (55)	20 (61)	12 (48)	13 (62)
	Cystic	7 (16)	4 (12)	4 (16)	2 (9)
	Glomerular	9 (20)	7 (21)	5 (20)	4 (19)
	Other	3 (7)	1 (3)	2 (8)	1 (5)
	Unknown	1 (2)	1 (3)	1 (4)	1 (5)
Stage of CKD	CKD3	2 (5)	1 (3)	1 (4)	1 (5)
	CKD4	7 (16)	5 (15)	4 (16)	5 (24)
	CKD5	14 (32)	12 (37)	10 (40)	7 (33)
	CKD5D	19 (43)	11 (33)	9 (36)	6 (29)
	PD	11 (52)	6 (18)	5 (56)	4 (67)
	HD	9 (43)	4 (12)	3 (33)	1 (16)
	HHD	1 (5)	1 (3)	1 (11)	1 (16)
	CKD5T	4 (8)	4 (12)	1 (4)	2 (9)
Phosphate	On binders	34 (77)	28 (85)	21 (84)	17 (81)
Binders	≥ 2 tablets/meal	15 (42)	14 (50)	-	-
Anthropometry					
Weight for age, Z score, mean (SD)		−0.67 (1.18)		-	-
Height for age, Z score, mean (SD)		−0.90 (1.10)		-	-
BMI for age, Z score, mean (SD)		−0.32 (1.37)		-	-
% weight for height, mean (SD)		98.7 (19.2)		-	-

BMI, body mass index; CAKUT, congenital abnormalities of the kidney and urinary tract; CKD, chronic kidney disease; ECH, Evelina London Children’s Healthcare; F, females; GOSH, Great Ormond Street Hospital for Children; HD, hemodialysis; HHD, home hemodialysis; M, males; NCH, Nottingham Children’s Hospital; PD, peritoneal dialysis; PUKA, phosphate understanding and knowledge assessment;.

a Other languages include Bengali, Pashto, Polish, Portuguese, Punjabi, Russian, Somali and Urdu.

**Table 2:** Routine baseline biochemistry

Laboratory parameter	Median (IQR)	*n*
Serum phosphate (mg/dl)	4.96 (4.09–5.39)	44
Serum phosphate (mg/dl) > 5.57		9
Serum phosphate (mg/dl)		
< 13 yrs	4.24 (3.65–5.08)	16
≥ 13 yrs	5.11 (4.30–5.57)	28
	< 13 yrs vs. ≥ 13 yrs	*P* = 0.01
Serum phosphate (mg/dl)	4.96 (4.02–5.39)	44
Serum calcium (mg/dl)	9.66 (9.02–10.34)	40
Parathyroid hormone (pg/ml)	176.3 (29.2–461.1)	40
Serum ionized calcium (mg/dl)	5.21 (4.41–5.21)	30
25 (OH)D (ng/ml)	32 (25–40)	42
Status (%)^18^		
Deficient		17%
Insufficient		31%
Sufficient		52%
Alkaline phosphatase (U/l)	178 (109–276)	44
Blood urea nitrogen (mg/dl)	47.4 (37.1–66.2)	44
CO_2_ (meq/l)	23 (20–25)	39

IQR, interquartile range.

Ionized calcium levels were not routinely measured at all centers.

### PUKA Questionnaire and Knowledge Scores

Median knowledge scores were significantly lower for children than caregivers (64.3% [55.3%–78.6%] vs. 72.7% [64.3%–85.7%], *P* = 0.04). There was a moderate correlation between the scores for child-caregiver dyads (*P* = 0.003, *r* = 0.50) (Table 3). Low-scoring questions included knowledge of side effects of hyperphosphatemia, identifying low phosphate foods, and understanding how P-binders work.

**Table 3:** Phosphate Understanding and Knowledge (PUKA) questionnaire completion rates and knowledge score results

Variable	All *N* = 77	Child *n* = 44	Caregiver *n* = 33	Child vs. caregiver
Use of QR code (%)	62	68	52	
Ease of completion (%)^a^	86	85	88	
Cronbach’s alpha (α)^b^		0.97	0.96	
Knowledge scores (% correct)				
Median (IQR)	71.4 (63.6–78.6)	64.3 (55.3–78.6)	72.7 (64.3–85.7)	*P* = 0.04
Lowest scoring multiple choice questions (% correct)				
If your blood phosphate levels are high, what might happen?		23	27	
Which foods contain very little phosphate?		31	46	
How do you think phosphate binders work?		43	48	
Child-caregiver dyads				
Knowledge scores (% correct)	All *N* = 60	Child *n* = 30	Caregiver *n* = 30	Child vs. Caregiver^c^
Median (IQR)	71.4 (57.1–81.8)	64.3 (44.7–77.1)	72.7 (64.3–85.7)	*r* = 0.5 *P* = 0.003

IQR, interquartile range.

a Self-rated score ≥ 4 stars out of total 5.

b Measured for 10 questions.

c Correlation between knowledge scores for child and caregiver within dyads.

**Figure 1:**
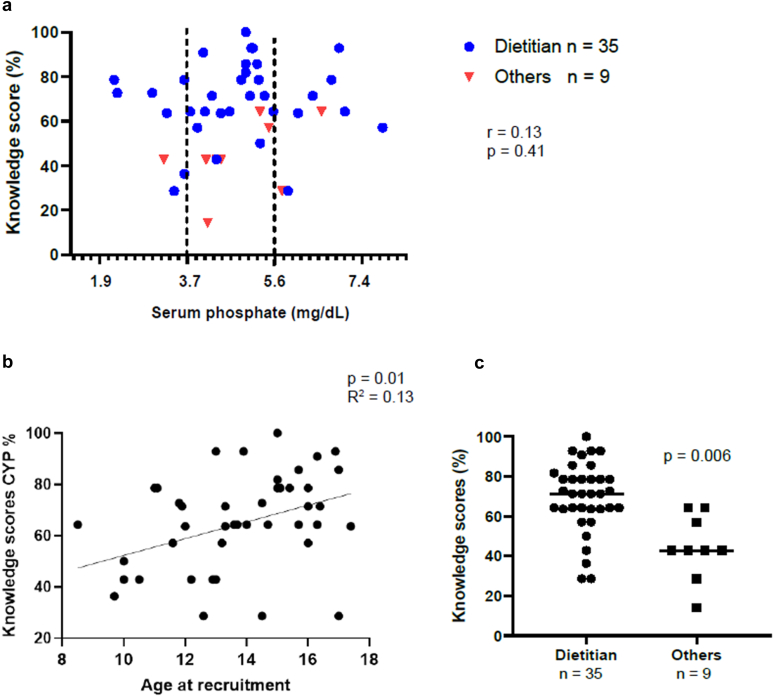
Knowledge scores versus serum phosphate for children, child age, and educator. (a) association between phosphate knowledge scores (% correct answers) in children with chronic kidney disease, serum phosphate levels and main educator (dietitian vs. others) (reference range for serum phosphate shown as 3.7–5.6 mg/dl). (b) association between knowledge scores in children with chronic kidney disease and age. (c) association between knowledge scores in children according to educator type.

Knowledge scores were not associated with serum phosphate levels (*P* = 0.41, *r* = 0.13,) (Figure 1a) or parathyroid hormone levels; however, there was a weak but significant association with age (*P* = 0.01, *R*^2^ = 0.13; Figure 1b). There was no association between knowledge scores and CKD stage, dialysis status, sex, or P-binder use. Children who had received education from a dietitian (80%) had a significantly higher knowledge score than those who received education from non-dietetic colleagues (including nurses and pediatric nephrologists) (*P* = 0.006; Figure 1c); however, there was no significant difference in serum-P between these 2 groups (*P* = 0.41; Figure 1a).

### Difficulties With Phosphate Management

Administering P-binders and/or following dietary phosphate modifications was difficult for 66% (35/53) of children and 94% (40/44) of caregivers. For 59% of children and 71% of caregivers, food restrictions posed the greatest challenge (Table 4). However, 39% of children and 29% of caregivers found P-binder use problematic (Table 4). Specific difficulties reported included remembering to take them in the context of polypharmacy (60% children, 50% caregivers); 53% disliking the taste; 40% children (50% caregivers) stated maintaining adherence outside of the home challenging; 33% said they made them nauseous; and 27% were put off eating. Twenty percent of children perceived that the binders made them feel different from others, contrasting with caregivers (38%).

**Table 4:** PUKA questionnaire results - difficulties experienced

Question	Child	Caregiver
**Do you find it difficult not being able to eat certain foods or having to take phosphate binders with your food?**	73% (32) Yes^a^	94% (31) Yes^a^
**Which of these do you find most difficult?**		
Not being able to eat certain foods	59% (19)	71% (22)
Having to take phosphate binder medication	13% (4)	13% (4)
Not being sure what I should be eating	13% (4)	13% (4)
Having to talk about what I eat	3% (1)	-
Everything	-	3% (1)
Nothing	9% (3)	-
Other (free text)	Life (1)	-
Do you have problems taking phosphate binders? Yes^a^	42% (15)	29% (8)
**Why is it difficult?** (allowed multiple responses)		
I have lots of medicines and can't remember to take them all	60% (9)	50% (4)
I forget to have them with me when out	40% (6)	50% (4)
I hate the taste	53% (8)	50% (4)
They put me off eating food	27% (4)	-
I can’t swallow them	7% (1)	13% (1)
They make me feel sick	33% (5)	25% (2)
I don’t want to seem different from my friends	20% (3)	38% (3)
I don’t understand why I have to take them	7% (1)	-
Other (free text)		-
They get stuck in my throat	2% (1)	-
I hate taking them	2% (1)	-
Feel too large		3% (1)
**Which of these could help them to remember to take your PHOSPHATE BINDER medicines with your food?** Helpful		
Putting a reminder on my phone	39% (14)	36% (10)
Putting medication in school lunch box	50% (18)	43% (12)
Keeping my medication in the kitchen at home	56% (20)	75% (21)
Giving spare medication to my school nurse	50% (18)	50% (14)
Keeping spare medication in my school bag	42% (15)	46% (13)

Percentage % (*n*).

a Includes “sometimes” and “I’m not sure”.

### Dietary Phosphate Education Experiences

Most education about the reduced phosphate diet was from a dietitian (80%); and 64% and 76% of children and caregivers, respectively, reported they understood the advice (Table 5). Despite this, most caregivers sought additional information online (88%). Their preferred delivery mode for education was direct communication, although written leaflets (diet sheets), food pictures, and games were also valued (Table 6). Further suggestions for educational content included meal ideas for home (61% children, 76% caregivers), school or college (39% children, 49% caregivers), and eating out (41% children, 67% caregivers). In total, 13 children and 11 caregivers made suggestions for improving education (Supplementary Table S1), which included pre-recorded cooking videos, animations, a mobile app, and more detailed information on dietary phosphate limits.

**Table 5:** PUKA questionnaire results - phosphate education

Question	Child	Caregiver		
Have you been taught which foods are high in phosphate?	89% (39)^a^ Yes	11% (5) Unsure	94% (31) Yes	6% (2) Unsure
**If you have been taught which foods are high in phosphate, who was this from?** Tick as many as you want				
A dietitian	80% (35)	97% (32)		
A doctor	34% (15)	30% (10)		
A nurse	20% (9)	18% (6)		
I'm not sure	9% (4)	-		
Parents	14% (6)	-		
Own research (on internet)	2% (1)	9% (2)		
How was this information given to you? Tick as many as you want				
Talking with a dietitian/nurse/doctor	91% (40)	94% (31)		
Getting an information sheet	36% (16)	73% (24)		
Looking at an app	14% (6)	15% (5)		
Getting recipes	14% (6)	15% (5)		
I'm not sure	5% (2)	-		
From parent	2% (1)	-		
“Food guide”	2% (1)	-		
Looking at labels shopping	2% (1)	-		
Internet search	-	3% (1)		
**Did you understand the information you were given about phosphate?**	64% (28) yes	36% (16)^b^ no	76% (25) yes	24% (8) some
**Have you tried to find information about phosphate from any of the following?** (PUKA-a only) Choose as many answers as you want				
Books	-	18% (6)		
Internet/computer	-	88% (29)		
An APP	-	6% (2)		
Social media	-	12% (4)		
Other families who have a child with kidney disease	-	15% (5)		
Kidney charities	-	21% (7)		
Not looked	-	9% (3)		

Percentage % (*n*).

a Or the family.

b Including “some of it” or “not sure.”

**Table 6:** PUKA questionnaire results – learning preferences

Question	Child	Caregiver
**How do you usually like to learn new things (such as which foods are best to eat)?** Choose the main one		
Someone (such as a dietitian) telling me what to eat	39% (17)	45% (15)
Looking at a written leaflet explaining what foods I can eat	14% (6)	9% (3)
Looking at pictures of what I can eat	11% (5)	15% (5)
Playing games or a quiz to help me learn	20% (9)	12% (4)
Talking with other children/young people	9% (4)	3% (1)
I don’t want to hear about what I should eat	5% (2)	9% (3)
The internet	2% (1)	-
Renal friendly recipes	-	3% (3)
Look at leaflet on my own – not in hospital setting	-	3% (3)
**What do you think would be helpful to teach you about foods that are high in phosphate?**		
A game	30% (13)	33% (11)
A list on the fridge or a fridge magnet	36% (16)	58% (19)
List of what to have in place of high phosphate foods	48% (21)	79% (26)
A picture guide	30% (13)	55% (18)
Regular chats with the dietitian	39% (17)	55% (18)
A quiz	23% (10)	15% (5)
An app	34%(15)	39% (13)
Video call to look at foods we normally eat at home	11% (5)	6% (2)
Supermarket trip to find out what I can eat	11% (5)	24% (8)
A short video to watch, which tells me what I can eat	27% (12)	30% (10)
An online teaching session with other children/young children	16% (7)	21% (7)
**What would help you to eat less food high in phosphate?** Tick as many as you want		
Meeting other children or young people who have to do the same thing	36% (16)	36% (12)
Having more ideas on what I can eat at home	61% (27)	76% (25)
Having more ideas on what I can eat at school/college	39% (17)	49% (16)
Having more ideas on what I can eat from a takeaway or in a restaurant	41% (18)	67% (22)
Nothing	2% (1)	-
I don’t need help	14% (6)	6% (2)
A quick reference app	-	3%(1)

Percentage % (*n*).

### Focus Groups

**Figure 2:**
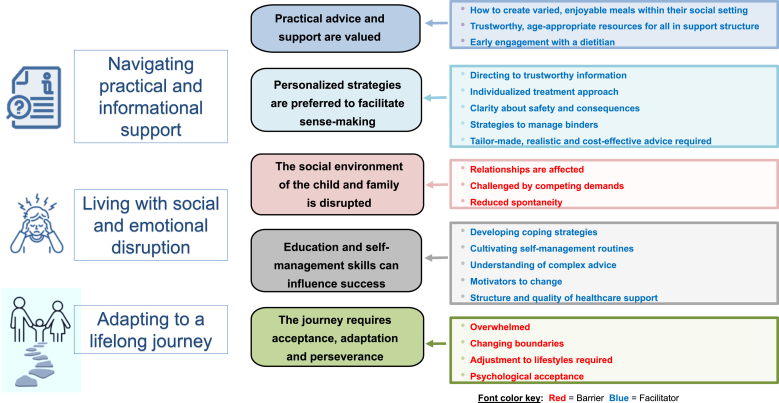
Experiences of phosphate management - overarching concepts, themes, and subthemes from focus groups.

Forty-six participants (25 children and 21 caregivers) took part in 13 online focus groups. Their age, sex, and ethnicity distributions were similar to those of participants who completed the questionnaire (Table 1). We identified 3 overarching concepts representing distinct but interconnected aspects of experiences of the phosphate management journey (Figure 2). These insights contribute to a deeper understanding of the origins of poor adherence. Exemplar quotes are provided in Supplementary Table S2.

#### Navigating Practical and Informational Support

*Practical Advice and* *Support are Valued*. Families emphasized the need for receiving clear, actionable guidance, when first introduced to the reduced phosphate diet. The diet was described as confusing, rigid, and monotonous, with limited snack options. Participants expressed a desire for more varied, enjoyable meals that fit within their social contexts. One caregiver shared *“We literally have the same diet week in week out and it is very boring*.*”* Decisions around food choices while shopping was also problematic. Participants highlighted the importance of professionals recommending trustworthy, age-appropriate, and accessible information to support all members of their network. Social media was used by children and families as a tool to facilitate learning and provide support.

Early engagement with dietitians was viewed as foundational to long-term adherence. Establishing a trusting relationship, consistency in messaging and personnel, and tailored dietetic support were identified as key factors for success: *“I think the engagement and the material that's provided is key at the start of the journey as to how successful that relationship is going to be with the dietitian and how compliant they're going to be moving forwards”* (caregiver). However, some resources provided were perceived as unsuitable or overly simplistic: *“Images are too childish”* (child, age 15 years).

*Personalized Strategies are Preferred to Facilitate Sense-Making.* As families progressed, they sought personalized strategies that reflected their unique circumstances. Many articulated a desire for access to safe, reliable and relevant information: *“You do learn stuff via the internet, but a lot of it isn't always trustworthy for your specific dietary requirements”* (child, age 16 years). Peer experiences were seen as a valuable mechanism to make sense of the journey *“I think maybe a video…how it has affected someone that's actually gone through it. Instead of, like, just someone telling you and they don't know how it really feels”* (child, age 14 years). Resources describing the negative side effects of hyperphosphatemia were not considered motivators to adherence in most children.

#### Living With Social and Emotional Disruption

*The Social Environment of the Child and Family is Disrupted by the Reduced Phosphate Diet.* Dietary restrictions disrupted family routines, social participation, and relationships. Eating out became difficult: *“We stopped eating out when we were told that I was not allowed to have large amounts of phosphate”* (child, age 15 years). Caregivers highlighted the need to balance dietary needs across siblings: *“If you’ve got more than 1 child…you don’t want the other one impacted, because one’s not allowed”* (caregiver).

The reduced phosphate diet imposed emotional, financial, and time burdens. Preparing separate meals was burdensome and exhausting: *“It's no fun making different meals all at the same time. Working parents find cooking for family challenging”* (caregiver).

Constant vigilance about food choice can lead to isolation, diminished spontaneity and enjoyment: *“becomes a stressful environment and you end up not wanting to go out and eat with your friends, because it just becomes a chore”* (child, age 16 years).

*Education and Self-management Skills can Influence Success*. Participants often conducted their own research before consulting health professionals: *“I did search my own questions. Then I went to the clinic and just confirmed with the doctor as well”* (child, age 17 years). Conflicting messaging from health care professionals created confusion and undermined confidence: *“There are conflicting messages coming from medical professionals…that’s when families get into difficulty”* (caregiver).

The adherence of children appeared to be shaped by their relationships with clinicians as well as the tone of messages or messaging: *“It’s just to keep the doctors and the clinic happy”* (child, age 13 years). Positive, motivational, framed messaging was preferred: *“You will feel better if you do this”* rather than fear-based warnings like *“You will end up in hospital if you don’t do this*.*”* Caregivers also felt that guidance from health care professionals helped reduce tension and resentment around food policing, ultimately improving adherence.

The link between education, self-management skills, and adherence was evident. Families valued clear, actionable guidance to support self-management, though developing routines around diet and binders was challenging. Binders were often hidden from peers or skipped during social occasions, reflecting the complexity of management in everyday life.

Participants described their strategies for success. One child shared how creativity in food preparation helped maintain enjoyment: *“You can still create something that tastes similar to it with stuff that you are allowed”* (child, age 13 years). Connection with peers facing similar challenges could enhance motivation and confidence: *”to share with other people that have had similar issues, she really would benefit”* (caregiver).

#### Adapting to a Lifelong Journey

Adherence appeared to be influenced by psychological readiness and acceptance. The early stages of the journey were often marked by confusion and frustration: “*You don’t know how much is a safe amount in foods*” (caregiver). Inconsistencies in dietary guidance contributed to emotional distress: *“I know what I’m supposed to have, but I’m also told, it’s not the worst thing if you do have it.”* (age 16 years). Successful adaptation was reflected in the overlapping themes from our focus groups, including the ability to build routines; develop coping strategies; and access to high-quality health care, family, and peer support.

Caregivers described a non-linear, gradual process of acceptance: “*He’s got to the stage where he’s accepted what he can and cannot eat*” (mother of a 9-year-old). However, some children continued to struggle with binders, particularly in social settings: *“If I’m by myself, I typically don’t take them because they make you really nauseous… and it’s sad, because I struggle enough with eating*” (age 16 years).

**Figure 3:**
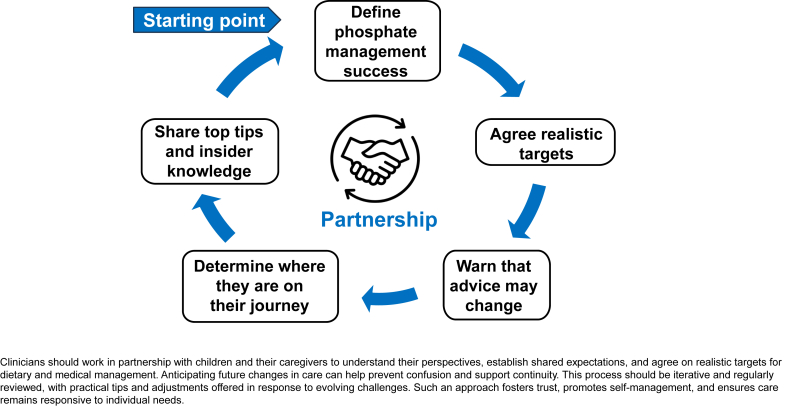
Suggested strategies for phosphate management in pediatric chronic kidney disease.

In addition, participants provided suggestions for clinicians to improve adherence, which informed the development of practical prompts (shown in Figure 3). These strategies will require evaluation in clinical settings to determine their effectiveness.

## Discussion

This is the first study to explore the real-world challenges of phosphate management in pediatric CKD from both the caregiver and child perspectives. The mixed-methods approach generated rich data to inform clinical practice and improve pediatric care. Key results included the lack of an association between knowledge scores and serum phosphate levels, and insights into pragmatic challenges associated with diet and P-binder adherence in children. Importantly, discrepancies between child and caregiver responses highlight the need to give children agency in describing their experiences with phosphate management, enabling the development of explicit, individualized self-management strategies.

This study found that 1 in 5 children had hyperphosphatemia, 78% of them in the 13 to 18 years age group. This is similar to data from the International Pediatric Peritoneal Dialysis Network Registry.^5^ Adolescents may be more resistant to infringements on their everyday life because of a mix of developmental, psychological, and social factors.^19^, ^20^, ^21^ Supporting adolescent autonomy through shared decision-making, non-judgmental dialogues (including discussion of possible barriers to adherence), self-monitoring and “SMART” goal setting may improve adherence.^22^ Balancing caregiver involvement may support the transition from externally imposed control to the development of internal motivation.

There is currently no universally accepted or validated threshold for determining what constitutes an adequate phosphate knowledge score to support behavioral change. In our study, the PUKA questionnaires were used to assess both factual understanding and the ability to apply this knowledge to everyday decisions. Although previous research has suggested a knowledge score ≥ 60% is representative of “sufficient knowledge” to support behavioral change,^23^ our findings showed no association between knowledge and serum phosphate, despite a median knowledge score of 64.3% for children and 72.7% for caregivers. This aligns with previous studies in both pediatric^24^, ^25^, ^26^ and adult^27^^,^^28^ populations. Lower knowledge scores have been reported in those aged 9 to 11 years in a previous study,^26^ which may reflect the traditional direction of education toward caregivers rather than children. In adult populations, phosphate knowledge is lower than that of salt, protein, or potassium,^29^^,^^30^ possibly reflecting the lower exposure to phosphate information in the public sphere.

The higher phosphate knowledge score in those taught by a dietitian is an informative and novel finding in our study. The reasons for this are unclear but may relate to the development of a trusted relationship with children and their families, along with the application of specialized professional skills. Periodically revisiting phosphate education with a dietitian is recommended, because food preferences and adherence are not static concepts, and similar to medication adherence,^31^ will vary over time. This recommendation contrasts with existing practice where dietetic advice is often only given when there is a deterioration in serum-P and/or when the young person starts to take ownership of their medical and dietetic care.

Results from the PUKA questionnaire show that most caregivers (88%) sought additional information online, suggesting they were seeking help beyond that given by the dietitian. The importance of guiding families toward reliable and trustworthy resources was a recurrent theme in the focus groups.

Knowledge regarding the consequences of a raised serum phosphate was suboptimal in this study of children, similar to reports in adults.^28^ This is surprising, because a recent review found 89% of phosphate educational materials used by dietitians include a description of these consequences.^7^ Insights from our focus groups suggested that children were more motivated by short-term quality of life benefits than long-term health risks, and adult research suggests that it may even have the opposite effect.^20^ This suggests a need to reframe adherence messaging. The lack of immediate recognizable or unpleasant symptoms associated with hyperphosphatemia may be relevant,^26^ resulting in an inability to apply the health belief model^32^ (behavior changes only occurring when the perceived benefits outweigh risks).

The interconnected themes identified in this study reflect the complex, multifaceted, and evolving journey of dietary phosphate management in pediatric kidney disease.^33^ Together, they illustrate how knowledge, context, and emotional resilience interact to shape the experiences of children and their families. Optimal phosphate control and adherence may only be achievable when the individual or their family are psychologically ready,^28^ a theme that was evident from our focus groups. Phosphate educational materials rarely contain actionable information,^7^ and our findings challenge the effectiveness of merely providing a diet sheet or a one-off teaching session to children and their caregivers. This approach risks underestimating the complexity of implementing changes within a family and social context. Interventions must be tailored to the family’s unique circumstances, must be realistic, and evolve over time in response to emerging challenges. Many focus group participants expressed a desire for the dietitian to take a more proactive role, moving beyond generalized information that can be difficult to interpret.

In most parts of the world, pediatric kidney dietetic staffing levels are insufficient, particularly for children with CKD stages 2 to 3.^8^ This lack of support may explain why some participants reported receiving contradictory dietary advice from different members of the health care team. Conflicting information can create confusion and distress for families, eroding trust in health care professionals and damaging the patient-clinician relationship. This breakdown in communication may compromise nutritional status, diminish enjoyment of food, and ultimately contribute to poor adherence to dietary advice.^34^, ^35^, ^36^, ^37^ Supportive interactions between patients and professionals were consistently cited as important by both children and caregivers. However, as has been reported in adults,^34^ many felt blamed by medical staff for hyperphosphatemia. Establishing strong, trusting relationships with the family unit takes time. This process involves understanding their expectations and needs, supporting them through everyday challenges, identifying barriers to adherence, and sharing insider knowledge. Children and their families identified several key motivators for adherence, including the child being directly addressed by medical staff during consultations, the provision of practical resources such as recipes, and support in developing sustainable routines.

An appreciation of the role of food in defining identity (with friends, community, and family) and relationships (between children and their caregivers),^19^ explains how imposing rigid dietary changes can lead to a feeling of isolation, resulting in a lower perceived quality of life. For young people acquiring social independence, the challenges of eating out were frequently mentioned and this has also been identified in adults.^19^ It is only by undertaking in-depth dietary consultations with a skilled professional that such issues can be appreciated and meaningful advice given.^19^

The findings of this study indicate that new strategies for phosphate management are required for this patient group (Figure 3). In adults with CKD, the use of a question prompt sheet before or during a consultation has been shown to be effective in improving diet quality.^37^ Building on this, our proposed strategies could be developed into a practical tool to support consistent and personalized dietetic care. A shared decision-making style approach is recommended, focusing on collaborative partnerships with families to define what success means to them, set realistic targets for dietary change and serum phosphate levels, and reinforce the importance of regular reviews, because kidney function and phosphate control may fluctuate over time. In addition, the development of resources using health literacy principles would support families in locating, understanding, and applying the health information provided.

This study provides rich insights into the challenges of phosphate management among participants from a wide range of ethnic backgrounds, nearly half of whom were on dialysis and 75% taking P-binders. The multicenter design enabled exploration of varied educational experiences, and the inclusion of 30 child-caregiver dyads allowed for comparison of perspectives. Another strength was the mixed-methods design and use of the PUKA questionnaire, facilitating timely access to relevant data to inform the focus group discussions. All members of the research team have extensive professional experience in managing children with CKD. This positionality may have influenced the framing of the questions for both the questionnaire and the focus groups, as well as the interpretation of participant responses (our reflexivity statement is presented in Supplementary Table S3). To mitigate potential bias, reflexive journaling and peer debriefing were employed throughout the study.

Limitations of this study include potential response bias because of the predominance of English-speaking participants. Although the PUKA has been translated into Dutch, French, German, Italian, and Turkish, it is unclear whether offering the questionnaire in additional languages would have increased participation. Another limitation is that completion of the questionnaire by children was unsupervised and may have been influenced by their caregivers.

Despite adequate dietary knowledge, improved phosphate control was not observed, suggesting that factors other than knowledge contribute to suboptimal adherence. This study clearly articulates the emotional, social, psychological, and financial burdens families face. Although practical advice and support are highly valued, this is not sufficient in isolation. Effective management requires a combination of practical tools, realistic and personalized strategies, and supportive environments to help families navigate the challenges of a reduced phosphate diet and P-binder use. The findings underscore that education and self-management skills can influence success, but only when embedded within a context that acknowledges the disruption to the child’s environment and the emotional journey of acceptance, adaptation, and perseverance. The next step is to place greater emphasis on shared decision-making and build strong partnerships between professionals and families, ensuring that future support packages are codesigned, accessible, and responsive to individual needs.

## Disclosure

All the authors declared no competing interests.

## Supplementary Material

Supplementary File (PDF) **Figure S1.** Study design. **Figure S2.** Focus group topic guide (children and young people). **Table S1.** Questionnaire responses outlining suggested strategies to support understanding of dietary phosphate among children and young people. **Table S2.** Themes and subthemes with exemplar quotes. **Table S3.** Reflexivity statement.

## References

[1] McAlister L., Pugh P., Greenbaum L. (2020). The dietary management of calcium and phosphate in children with CKD stages 2–5 and on dialysis-clinical practice recommendation from the Pediatric Renal Nutrition Taskforce. Pediatr Nephrol.

[2] Lambert K., Mullan J., Mansfield K. (2017). An integrative review of the methodology and findings regarding dietary adherence in end stage kidney disease. BMC Nephrol.

[3] Van Camp Y.P., Vrijens B., Abraham I., Van Rompaey B., Elseviers M.M. (2014). Adherence to phosphate binders in hemodialysis patients: prevalence and determinants. J Nephrol.

[4] Apostolou A., Karagiozoglou-Lampoudi T. (2014). Dietary adherence in children with chronic kidney disease: a review of the evidence. J Ren Care.

[5] Borzych D., Rees L., Ha I.S. (2010). The bone and mineral disorder of children undergoing chronic peritoneal dialysis. Kidney Int.

[6] Khan M.B., Carnethon M.R., Isakova T., Wolf M., Gutiérrez O.M. (2025). Effects of lowering dietary phosphorus additive intake on mineral metabolism in adults with and without chronic kidney disease. Clin J Am Soc Nephrol.

[7] McAlister L., Shaw V., Shroff R. (2024). Dietary phosphate educational materials for pediatric chronic kidney disease: are confused messages reducing their impact?. J Ren Nutr.

[8] McAlister L., Shaw V., Shroff R. (2025). 17 Phosphate education for paediatric chronic kidney disease: do renal dietitians consider educational resources are adequate?. BMJ Paediatr Open.

[9] Collinson A., McMullan M., Tse W.Y., Sadler H. (2014). Managing serum phosphate in haemodialysis patients: time for an innovative approach?. Eur J Clin Nutr.

[10] Malterud K., Siersma V.D., Guassora A.D. (2016). Sample size in qualitative interview studies: guided by information power. Qual Health Res.

[11] McAlister L., Shaw V., Pugh P. (2025). Development of a questionnaire to assess phosphate knowledge in children with chronic kidney disease and their caregivers. J Hum Nutr Diet.

[12] Bender D.E., Ewbank D. (1994). The focus group as a tool for health research: issues in design and analysis. Health Transit Rev.

[13] Horner S.D. (2000). Using focus group methods with middle school children. Res Nurs Health.

[14] Tong A., Sainsbury P., Craig J. (2007). Consolidated criteria for reporting qualitative research (COREQ): a 32-item checklist for interviews and focus groups. Int J Qual Health Care.

[15] Braun V., Clarke V. (2006). Using thematic analysis in psychology. Qual Res Psychol.

[16] Braun V., Clarke V. (2013).

[17] Cronbach L.J. (1951). Coefficient alpha and the internal structure of tests. Psychometrika.

[18] Shroff R, Wan M, Nagler EV (2017). Clinical practice recommendations for native vitamin D therapy in children with chronic kidney disease Stages 2-5 and on dialysis. Nephrol Dial Transplant.

[19] Christensen K.M., Bauer E.H., Prinds C. (2024). Exploration of low-phosphate diet management of patients receiving renal dialysis: an interpretive description. J Ren Care.

[20] Jin J., Sklar G.E., Min Sen Oh V., Chuen Li S., Min Sen oh V., Chuen li S. (2008). Factors affecting therapeutic compliance: a review from the patient’s perspective. Ther Clin Risk Manag.

[21] Greenley R.N., Stephens M., Doughty A., Raboin T., Kugathasan S. (2010). Barriers to adherence among adolescents with inflammatory bowel disease. Inflamm Bowel Dis.

[22] Hooker S., Punjabi A., Justesen K., Boyle L., Sherman M.D. (2018). Encouraging health behavior change: eight evidence-based strategies. Fam Pract Manag.

[23] Karavetian M., Abboud S., Elzein H., Haydar S., de Vries N. (2014). Nutritional education for management of osteodystrophy (NEMO) trial: design and patient characteristics, Lebanon. Nutr Res Pract.

[24] Ahlenstiel T., Pape L., Ehrich J.H., Kuhlmann M.K. (2010). Self-adjustment of phosphate binder dose to meal phosphorus content improves management of hyperphosphataemia in children with chronic kidney disease. Nephrol Dial Transplant.

[25] Blaszak R.T., Mitsnefes M.M., Ilyas M., Salman S.D., Belcher S.M., Brady D.R. (2005). Hyperphosphatemia in children receiving peritoneal dialysis--an educational program. Pediatr Nephrol.

[26] Abercrombie E.L., Greenbaum L.A., Baxter D.H., Hopkins B. (2010). Effect of intensified diet education on serum phosphorus and knowledge of pediatric peritoneal dialysis patients. J Ren Nutr.

[27] Schneider S.T., Klug A., Andrade J.M. (2024). Phosphorus knowledge and dietary intake of phosphorus of US adults undergoing dialysis. Nutrients.

[28] Durose C.L., Holdsworth M., Watson V., Przygrodzka F. (2004). Knowledge of dietary restrictions and the medical consequences of noncompliance by patients on hemodialysis are not predictive of dietary compliance. J Am Diet Assoc.

[29] Pollock J.B., Jaffery J.B. (2007). Knowledge of phosphorus compared with other nutrients in maintenance dialysis patients. J Ren Nutr.

[30] Cupisti A., Ferretti V., D’Alessandro C. (2012). Nutritional knowledge in hemodialysis patients and nurses: focus on phosphorus. J Ren Nutr.

[31] Horne R., Weinman J., Barber N. (2005).

[32] Janz N.K., Becker M.H. (1984). The health belief model: a decade later. Health Educ Q.

[33] Kalantar-Zadeh K. (2013). Patient education for phosphorus management in chronic kidney disease. Patient Prefer Adherence.

[34] Forfang D., Edwards D.P., Kalantar-Zadeh K. (2022). The impact of phosphorus management today on quality of life: patient perspectives. Kidney Med.

[35] Lambert K., Mansfield K., Mullan J. (2019). Qualitative exploration of the experiences of renal dietitians and how they help patients with end stage kidney disease to understand the renal diet. Nutr Diet.

[36] Hollingdale R., Sutton D., Hart K. (2008). Facilitating dietary change in renal disease: investigating patients’ perspectives. J Ren Care.

[37] Lambert K., Tulissio N., Cosier D. (2024). Impact of a health literacy sensitive model of care in outpatient nephrology dietetic clinics. J Hum Nutr Diet.

