# Supplementary material for: Barriers to and Facilitators of Phosphate Control in Children With CKD
Source: Kidney Int Rep. 2025 Oct 10;10(12):4252–63. doi: 10.1016/j.ekir.2025.09.045 (PMC12712525; doi:10.1016/j.ekir.2025.09.045)
Supplement: Supplementary File (PDF) — Figure S1. Study design. Figure S2. Focus group topic guide (children and young people). Table S1. Questionnaire responses outlining suggested strategies to support understanding of dietary phosphate among children and young people. Table S2. Themes and subthemes with exemplar quotes. Table S3. Reflexivity statement. [file mmc1.pdf]

Supplementary Material

Table of contents:

Supplementary Figure 1.	Study design
Supplementary Figure 2.	Focus group topic guide (children and young people)
Supplementary Table 1.	Questionnaire responses outlining suggested strategies to support understanding of dietary phosphate among children and young people
Supplementary Table 2.	Themes and sub-themes from the focus groups with exemplar quotes
Supplementary Table 3.	Reflexivity statement

Supplementary Figure 1. Study design

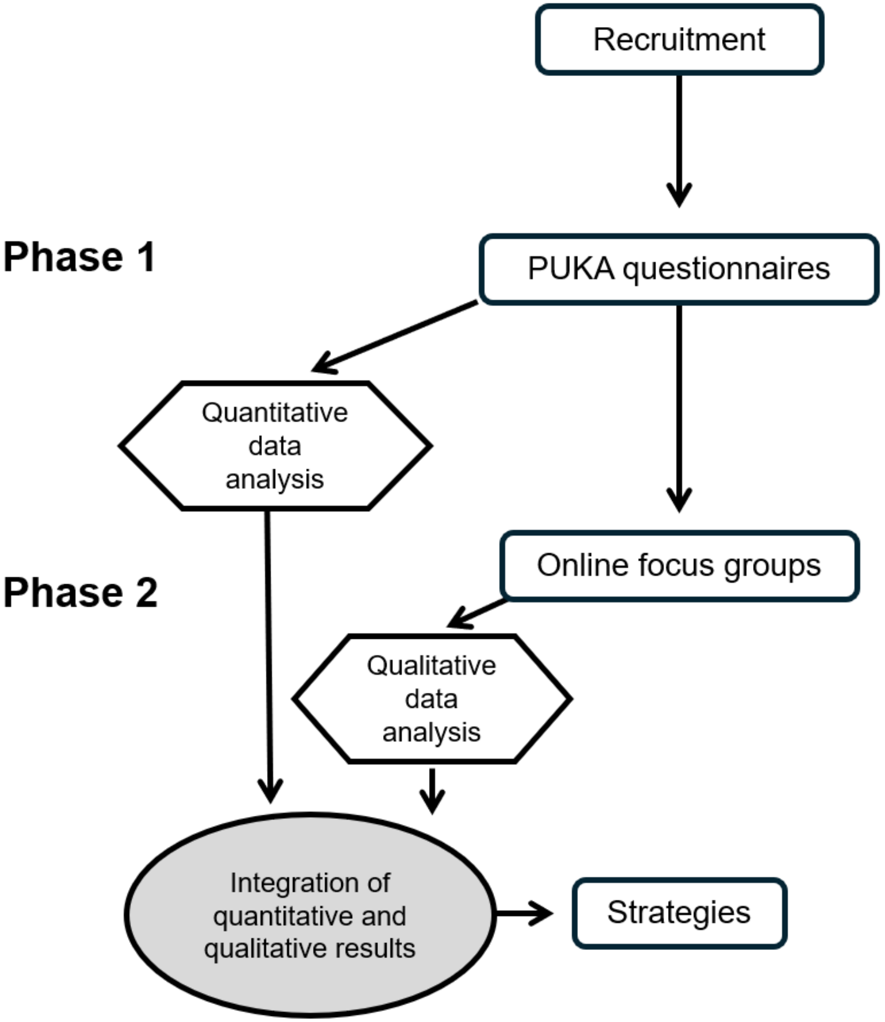

INCLUSION

Children and young people with chronic kidney disease (CKD) and one caregiver

CKD stages 4-5D or recent transplant (<6 months)

Aged 8-18 years
3 United Kingdom kidney units

Baseline biochemistry
Demographics

Knowledge scores

Coding
Inductive thematic analysis

EXCLUSION

Neurodevelopmental or cognitive disorders

or phosphate-losing conditions (tubular disorder)

or intensive home or in-center hemodialysis

or fully enterally tube fed

or limited spoken or written language skills

Supplementary Figure 2. Focus group topic guide (children and young people)

GREAT ORMOND STREET INSTITUTE OF CHILD HEALTH
IMPACT STUDY
Great Ormond Street Hospital for Children NHS Foundation Trust

FOCUS GROUP TOPIC GUIDE (children and young people)

GENERAL INSTRUCTION Before turning on the recorder: *check battery*

> **Standard information to give if no understanding of phosphate:**
>
> Phosphate is present in food and is important for the development of strong bones and to produce energy for the body. Our kidneys keep the level of phosphate safe by getting rid of any excess in the urine (wee). When your kidneys are not working properly, the phosphate levels in your blood may increase.

Introduction

Hello everyone and thank you all so much for joining me today

Introduce yourself

- Good morning. So, my name is X and you may know me as I work as a part of the team (as a dietitian) at X looking after children and young people with kidney problems. I also have X with me, who will be taking notes if that is OK, as I want to remember everything we chat about.
- Today – I am not here to teach you anything! I want to learn from you.

So why are you here?

- I am doing some work to try and understand the best way of helping you, and other young people like you and your families to make the changes to what you eat needed because of your kidney problems

You have been invited because:

- You all have problems with your kidneys
- We have invited CYP aged 8 or above who go to hospitals in this country including GOSH in London but also *Newcastle and Nottingham*, (and also their caregivers) to help us with this.

What is going to happen today?

This session will have 2 parts:

For the first:

- I would like to ask you all a few questions about food and how you feel about some of the changes you may have been asked to make to what you eat and the medicines you might have to take with food.

For the second:

- I want you to tell me what you think about different ways that dietitians and doctors have tried to help children with kidney problem understand how to make these changes.

Grounds rules

- I really want to learn and hear from all of you. There are no right or wrong answers.
- Do listen to each other and talk to each other. Please comment on what others are saying.
- You don't have to chat if you don't want to. Some of you may have a PADDLE stick with a thumbs up and down on it, you can wave that if you want to agree or disagree. We will see how we get on with our microphones on mute unless you are speaking if you are able to do that.

Opening (warm up) question

"So, before we start with our main questions"

Would you like to tell us your name and tell us something that is **important to you** – for example: friend, pets, family, TV shows, computer games or foods.

Starting and confidentiality

- You have **already said you are happy** to help us today and signed a form along with your parents – are you still OK with this?
- I am going to record this session as we have previously discussed – is that still ok?
- These recordings are only used this for research, and nobody will know who said what – your name will not be alongside any comments you make.

TURN ON RECORDER

IMPACT study focus group topic guide CYP

GREAT ORMOND STREET INSTITUTE OF CHILD HEALTH
IMPACT STUDY
Great Ormond Street Hospital for Children NHS Foundation Trust

FOCUS GROUP TOPIC GUIDE (children and young people)

CORE QUESTIONS

Opening question:

1. I want to talk about foods. Are there any foods that you are not meant to eat?

Prompts: Do you know why?

2. Have any of you heard about something called phosphate?

Prompts:

- What has this to do with your body? Is it in your body /food?
- Does it have anything to do with your kidneys?
- Do you remember who talked about this with you? How did that make you feel?
- Can you remember what they said were the **problems for your body** with high phosphate levels

3. Can you tell me any food or drinks that you think may be high in phosphate?

Prompts:

- Give me some examples of drinks?
- Can you think of any snacks that are high in phosphate?
- How **can you tell if a food** is high in phosphate?
- Do you know any foods that are low in phosphate?

4. Do you eat or drink any of this food high in phosphate eg milk or eat cheese, chocolate, eggs?

Prompts:

- Who decides what you eat?
- If you do not eat these – do you miss these? What do you miss most?
- Some people find it difficult to avoid certain foods, what about you? Is this more difficult at certain times? (eg at school, at a friend's house, when they have a takeaway?)

5. How would you like to be told (taught) about what you should eat?

Prompts:

- Chatting with someone at a hospital; Video consults with a dietitian
- Booklets – style/colour – pictures
- Fridge lists
- An app – what should it say? What should it help you with?
- Recipes
- Videos – Youtube
- Recipes
- Games

6. Do any of you take a chalky medicine or a tablet/powder (called a phosphate binder) when you eat?

Prompts:

- What is that like?
- What do you think about them?
- Do you know what it does?
- How do you remember to take them?
- What foods do you take it with?
- How do you carry it to school?
- What advice would you give other people on how to take these

Show slides 1-8

Slide 1

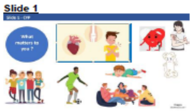

IMPACT study focus group topic guide CYP

GREAT ORMOND STREET INSTITUTE OF CHILD HEALTH
IMPACT STUDY
Great Ormond Street Hospital for Children NHS Foundation Trust

FOCUS GROUP TOPIC GUIDE (children and young people)

These are some pictures of some of the side effects of a high blood phosphate level.

Prompts:

- What do you think they are telling you?
- Do you mind if your bones hurt or get itchy skin or red eyes or long term problems with your heart or bones? What actually matters to you?

Introducing slide 2:

- Have you ever looked at the booklets or online stuff we have given your family? What would make you look at it?
- We tend to give you leaflets either in a clinic or via email or electronically. Would you prefer just to chat with a dietitian about what you have to do....or do you like having a written or pictorial printed guide to remind you what we said or watch an animation, video, play a game, read a book.

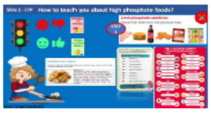

Slide 2

- Are you aware of the idea of traffic lights – what do they mean?
- People have used this to help people understand which foods are not good (so STOP/avoid or red) if you are trying to eat less phosphate, Amber (orange – control how much you eat – often have a daily or weekly amount you should keep to), Green (GO – allow freely as low in phosphate).

Prompts:

- The big RED or STOP is phosphate additives – have you heard of these?
- Why are they a worry?
- What do you think of some of these pictures.

Slide 3

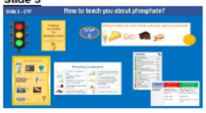

Prompts – introduce different "medium" examples

- The middle traffic light is the orange or amber one. You can have these foods but in lower amounts as they are high in phosphate. Do you think these pictures show this?
- Some dietitians produced swapping list – like this one. So, you could be told you could have 3 portions per day. Is that helpful?
- Here is a list showing high and low phosphate foods is that clear?
- You could be able to look up exactly how much phosphate it is has in it and avoid those with the most and have less of those with a medium amount.
- What do you think of these pictures?
- Do you prefer one than another? Why is that?
- Do you prefer the idea of avoiding something completely or being allowed smaller portions.

Slide 4

What do you this this is telling you?

It is called the phosphate pyramid and they use this in some other countries to help adults and children understand the foods high in phosphate. Is this helpful?

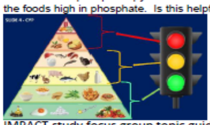

IMPACT study focus group topic guide CYP

GREAT ORMOND STREET INSTITUTE OF CHILD HEALTH
IMPACT STUDY
Great Ormond Street Hospital for Children NHS Foundation Trust

FOCUS GROUP TOPIC GUIDE (children and young people)

- Have you heard about encouraging more vegetables and plant-based foods?

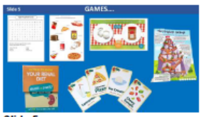

Slide 5

Would you be interested in playing any games to help you learn about phosphate?

(Talk through the games on the slide)

- Have you seen any of these?
- Can you think of a better game?

Slide 6

Recipes

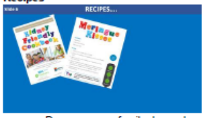

- Do you or your family do much cooking at home or do you mainly buy in readymade meals for heating up?
- Would you like to learn more about cooking? What would you like to know how to cook?
- Do you like the colours of this booklet?

Slide 7 – play a bit of video

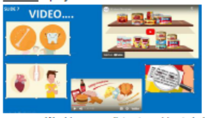

- Would you ever listen to a video to help you understand your diet. Im going to play you a bit of this one – what do you think?

Slide 8

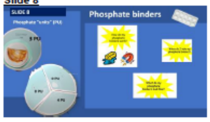

- Discuss how they think binders work
- Is this way of teaching in "phosphate units" to judge how many binders to take with foods?
- What would you like to tell other CYP about taking binders.

Closing section:

- Thank you all so much.
- What advice would you give other children or young people on how to make some of the dietary changes to protect their kidneys?
- Is there anything you would like to tell me about? Do you have any questions for me?

Summarise their comments/discussion if time permits

IMPACT study focus group topic guide CYP

Supplementary Table 1. Questionnaire responses outlining suggested strategies to support understanding of dietary phosphate among children and young people

Do you have any other ideas of ways to help you or other children and young people understand about foods that are high in phosphate?

Child (n 13)	Caregiver (n 11)
- Leaflets/posters	- Food sheets with list of foods
- A short reality/ drama programme for 13-16 yrs	- Talk to other young people with kidney problems.
- Find alternatives for that certain food	- Teach children about healthy home cooked foods
- Use apps or games for young children to understand better	- Fun animated food cartoon / comic, similar to 'annoying Orange' or 'Teen titans'
- Getting taught in schools	- An improv style video discussion, similar to the 'Made in Chelsea' or 'Next step', that is based around eating
- Tell us an estimate on how much of the banned foods we can eat	- Being taught about this at school
- Listen to your mum about what you can and can't eat every day. They are helping you.	- Educating them and their parent/s, with information given to them in a way they can easily process in their own time and refer back to any time
- A list of foods that are high in phosphate / that should be avoided	- Online pre-recorded cooking videos discussing phosphate and demonstrating how to prepare snacks low in phosphate.
- Make sure to listen to the doctors, nurses, dietitians	- Short videos, leaflets
- An app	- An app that would utilize the bar codes on packaging & search bar for everything else. This could have a rating system on whether to avoid or not to worry
- Pictures you could look at and sort them into groups- high in phosphate, low in phosphate. This could have a chart with color coordinated graphs showing children what is good or not good for them to eat	- Computer game or an app
- Eat what your dietitian told you	

Supplementary Table 2. Themes and subthemes from the focus groups with exemplar quotes

NAVIGATING PRACTICAL AND INFORMATIONAL SUPPORT	
**PRACTICAL ADVICE AND SUPPORT ARE VALUED**	**Source**
**How to create varied, enjoyable meals within their social setting**	
“The difficulty is finding variety but particularly when we all want to join in the same meal”.	Father of a 16-year-old girl
“Spend about ten minutes standing in the middle of an aisle trying to figure out if I should buy it or not”.	16-year-old girl
“We literally have the same diet week in week out and it is very boring”.	Father of an 8-year-old girl
**Trustworthy, age-appropriate resources for all in support structure**	
“You do learn stuff via the internet, but a lot of it isn't always trustworthy for your specific dietary requirements”.	16-year-old girl
“Websites will have conflicting information”.	16-year-old girl
“Images are too childish”.	15-year-old girl
“I feel like the leaflets that I was given in hospital, I had a quick look at them, that was it. I didn't really think much of them”.	16-year-old girl
“Almost everyone our age is on social media and it's such an accessible thing, and then rather than having to explain to my friends over and over ‘no, I can't have this but I can have this’, and they're asking me questions that I don't know how to answer, I can just link them to a page and they can learn about it themselves”.	16-year-old girl
**Early engagement with a dietitian**	
“I think the engagement and the material that's provided is key at the start of the journey as to how successful that relationship is going to be with the dietitian and how compliant they're going to be moving forwards”.	Mother of 13-year-old boy
**PERSONALIZED STRATEGIES ARE PREFERRED TO FACILITATE SENSE-MAKING**	
**Signposting to trustworthy information**	
“They want the information, not the games”.	Father of a 14-year-old girl
“You can't take people's word for something as a given. You, kind of, have to still research it because obviously, that's their opinion and their recipe that they've made up or found somewhere else”.	Mother of an 11-year-old boy
“You do learn stuff via the internet, but a lot of it isn't always trustworthy for your specific dietary requirements, because not everyone is restricted on all of the danger groups as per se and some people are more heavily restricted than others. So, you just have to get to know your own diet”.	16-year-old girl
**Individualized treatment approach**	
“My mum was a really big help, she'd always say before dinner, 'Take this,' or, 'Do that,' but I mean, I knew as well, but sometimes before dinner, I wouldn't remember.. if she wasn't around	13-year-old boy
“I'd bring them into school, or sometimes, I'd hand them into reception, and then, I'll go to a certain time and just take them”.	13-year-old boy
**Clarity about safety and consequences**	
“Sometimes because she was eating-, or she could eat, like, a 500ml tub of yogurt without even breaking sweat, things like that, and then if it didn't ever come up in the bloods then I'd find things getting more and more lapse”.	Father of a 14-year-old girl
“I think because she doesn't understand the benefits of it fully, fully the benefits of it, she doesn't see it as a priority”.	Father of a 16-year-old girl
“I think maybe a video because then you can see, like, how it has affected someone that's actually gone through it. Instead of, like, just someone telling you and they don't know how it really feels”.	14 year-old girl

**Strategies to manage binders**	
"The crushing method works. It worked for us and we just put it in a little bit of water and he just took it".	Mother of a 9-year-old boy
"If the medicine is kind of shown to the child as a super-hero ..then the child kind of believes in it and starts taking it on their own rather than having a battle".	Mother of a 17-year-old boy
"If you put that under your tongue and not just straight on your tongue, then you can swallow it easier, because if it goes on your tongue, you might start feeling it dissolved, and then, it doesn't taste very nice".	13-year-old boy
"I practice with Tic Tacs, like those little sweets".	10-year-old boy
**Tailor-made, realistic and cost-effective advice required**	
"So, I think there could be, maybe, something there, but I think it's all tailor-made, isn't it? You can't. What works for one person isn't going to work for another, and that's probably where you struggle, as dieticians, in trying to give the information".	Mother of a 13-year old boy

LIVING WITH SOCIAL AND EMOTIONAL DISRUPTION

THE SOCIAL ENVIRONMENT OF THE CHILD AND FAMILY IS DISRUPTED

**Relationships are affected**	
"My family used to moan all the time that, 'This food is disgusting' ".	12-year-old girl
"My siblings ask for a takeaway and my parents go, 'No, we can't because of your older sister. She can't really have anything'".	11-year-old girl
"When he goes, maybe, to family member's houses…he gets a little bit upset if they are having, maybe, a particular kind of food and he can't have it".	Mother of a 9-year-old boy
"My family were coping with it as well, but I'd tell them, 'It's okay, you can eat around me, like, the stuff that I'm not allowed to eat."	Mother to a 13-year-old boy
**Challenged by competing demands**	
"You know, you buy crumpets, the phosphate ones are much cheaper. The one's without the phosphate are much more expensive, and obviously, you've got cost of living as well".	Mother of a 13-year-old girl
"If you've got more than one child, like we all have, you don't want the other one impacted, because one's not allowed".	Mother of a 13-year-old boy
**Reduced spontaneity**	
"Stopped eating out when we were told that I was not allowed to have large amounts of phosphate".	15-year-old girl
"You're constantly having to read packets".	Mother of a 10-year-old boy
"If you're out with friends and everyone's trying to decide where to eat, ….just becomes a stressful environment and you end up not wanting to go out and eat with your friends, because it just becomes a chore".	16-year-old girl

EDUCATION AND SELF-MANAGEMENT SKILLS CAN INFLUENCE SUCCESS

**Developing coping strategies**	
"The first thing to do is to just Google it and see what comes up".	Mother of an 11-year-old boy
"I make the decisions because I know what I can eat, what I can't eat. It's quite easy when you've been doing it for the last two yrs of your life".	16-year-old boy
"You can still create something that tastes similar to it with stuff that you are allowed"	13-year-old boy
"Sometimes obviously you lie, for example, if I'm out with my friends, I'm not just going to get it out and take it, like, I just don't like the way it looks because it's in a big box. Obviously, I'm not going to take that out wherever I'm going. I just don't eat when I'm out with my friends".	16-year-old boy

"Just try and find substitutes that you do like. Like, you don't have to have food you don't like because it's similar to the food you used to be able to have, try and find nice substitutes".	14-year-old girl
**Cultivating self-management routines**	
"I then put them in, like, a container that I put in my pocket, then I couldn't forget, because they're constantly there rattling".	13-year-old boy
"Some of my friends were a big help. Like, some of them would be like, 'Oh, don't you have to go and take meds?' and I'm like, 'Oh, yes, I just forgot,' and then I'd go do it, but yes, it was, I mean, I'm not ashamed of having to take them or anything, it's not a bad thing. So, I don't mind sharing".	13-year-old boy
"(To) some of my friends, like, I said, 'Oh, it's just stomach stuff,' and they just went, 'Okay, yes, cool' ".	14-year-old girl
"If I see the word phosphate, I know it has phosphate. Or if I see one of the E's, and then you search up to make sure it doesn't, like, have the phosphate, because I'm not sure if all the E's mean they have phosphate, like, I think, I'm not really sure".	15-year-old boy
**Comprehension of complex advice**	
"Infographics are really helpful to just be really clear".	16-year-old girl
"….talked to my parents about it and then when I showed more interest in learning ….they then started talking to me about it as well"	14-year-old girl
"I did search my own questions. Then I went to the clinic and just confirmed with the doctor as well".	17-year-old girl
"I found it easier to restrict on things like protein when I was given an amount of portions that I could have, and they said, okay, you can have so many portions of protein a day and this is what a portion looks like.. If I had been told that for phosphate, it probably would have been easier for me to manage it".	16-year-old boy
**Motivators to change**	
"I just, kind of, woke up feeling poorly and I had itchy skin quite a lot".	13-year-old girl
(Advice should) "be presented in a positive way rather than in an, initially, negative way. Saying, 'You'll become unwell,' et cetera. I think it has to be positive things first".	Mother of a 13-year-old girl
"I think it is good to talk about the reality because it will be a long, long ended battle but that's just my experience and I feel once I did tell him the reality it was a switch and now he takes his medicine and he reminds me now. It's not me reminding him. He will say, 'I need to take my medicine.' And I was like, 'Oh okay, that's right'".	Mother of a 9-year-old boy
"I just feel like showing examples of, like, what happens to the bones and heart and stuff is just better than saying that you'll feel rubbish and stuff".	15-year-old boy
**Structure and quality of health care support**	
"There are conflicting messages coming from medical professionals and you as a family, that's when families get into difficulty".	Mother of a 13-year-old boy
"It's better coming from the hospital professionals than from the parent because I think-, well, in my case, she respects a little more what they say and there's that separation of relationship".	Mother of a 17-year-old boy
"It was just, like, a big thing for me to keep the doctors and the clinic happy".	13-year-old boy
"We've been doing this journey for 12 yrs and it just, you know, it's grown with us from day dot the milk that they had and, you know, that it was just follow your lead, really".	Mother of a 12-year-old boy
**ADAPTING TO A LIFELONG JOURNEY**	
**THE JOURNEY REQUIRES ACCEPTANCE, ADAPTATION AND PERSEVERANCE**	
**Overwhelmed**	
"We looked like lunatics in the supermarket reading everything. Ham was the one that just sent her over the edge".	Mother of a 16-year-old girl
"You might as well tell them that they can never have the Wi-Fi password again because the world literally ends".	Mother of a 16-year-old girl
"You just sit there Googling everything really and then end up driving yourself nuts with it".	Mother of a 16-year-old
"He absolutely loved milk and he absolutely loved cheese and he absolutely detested any replacements that we gave him with a passion".	Mother of a 15-year-old boy

"You don't know how much is a safe amount in foods so you tend to get very bewildered by the ingredients on the labels".	Mother of a 16 year-old girl
**Changing boundaries**	
"We were actually we were told at one point to not be so strict and so diligent with the renal diet because actually his bloods were too good, they were dropping too low".	Mother of a 13 year-old boy
" 'Oh, but he can have this as a treat and you can have this as a treat, and a pack of crisps is okay.' And we're, like, 'Don't.' You know, it's conflicting messages, so, actually there needs to just be that level playing field that if they're on the renal diet they're on it and actually there's no-, because I think this is where it becomes difficult for families because where are those perimeters".	Mother of a 13-year-old boy
"I know what I'm supposed to have, but I'm also told, it's not the worst thing if you do have it. So, sometimes I just don't really care".	16-year-old boy
**Adjustments to lifestyles required**	
"It's no fun making six different meals all at the same time. Working parents find cooking for family challenging".	Mother of a 16-year-old boy
"I do have an older sister, but she enjoys take-out, but she also doesn't mind, you know, cooking and eating home-cooked stuff. So, it was fine for everyone, really".	15-year-old boy
**Psychological acceptance**	
"Lucky to have them diagnosed early".	Mother of a 12-year-old boy
"I mean he does know what he can and cannot eat and for the past year or so he is making an attempt himself to eat the right food".	Mother of a 17-year-old boy
(She) "goes through phases where she's accepting of diet and then she will also go through phases where she will really push against diet and I think that's just her trying to assert some control over the situation that she's in".	Mother of a 13-year-old girl
"He's got to the stage where he's accepted what he can and cannot eat….., a lot more knowledgeable in the sense of what he can and cannot eat and the impact it has on his health".	Mother of a 9-year-old boy
"Just get on with it".	16-year-old boy

Supplementary Table 3. Reflexivity statement

The main researcher (LM) is an experienced pediatric renal dietitian. The research was undertaken to improve the effectiveness of dietary advice. An assumption held by LM prior to the study was that poor adherence to P-management was due to the low quality of resources used to support education. LM undertook training in qualitative research methodology to lead focus group discussions. Focus group transcripts were coded independently by LM, VS, CH, PP and FB, under the guidance of KL. LM and KL have personal experience in living with a child with a chronic medical condition, so shared some of the experiences of the participants.
